# Appropriateness for SARS-CoV-2 vaccination for otolaryngologist and head and neck surgeons in case of pregnancy, breastfeeding, or childbearing potential: Yo-IFOS and CEORL-HNS joint clinical consensus statement

**DOI:** 10.1007/s00405-021-06794-6

**Published:** 2021-04-15

**Authors:** Alberto Maria Saibene, Fabiana Allevi, Tareck Ayad, Tomislav Baudoin, Manuel Bernal-Sprekelsen, Giovanni Briganti, Sean Carrie, Per Cayé-Thomasen, Sara Dahman Saidi, Nicolas Dauby, John Fenton, Wojciech Golusiński, Ludger Klimek, Andrée-Anne Leclerc, Yves Longtin, Giuditta Mannelli, Miguel Mayo-Yáñez, Cem Meço, Osama Metwaly, François Mouawad, Kazimierz Niemczyk, Ulrik Pedersen, Krzysztof Piersiala, Jan Plzak, Marc Remacle, Nathalie Rommel, Hesham Saleh, Dawid Szpecht, Miroslav Tedla, Camilla Tincati, Manuel Tucciarone, Karol Zelenik, Jerome R. Lechien

**Affiliations:** 1 Otolaryngology Unit, ASST Santi Paolo E Carlo, Department of Health Sciences, Università Degli Studi Di Milano, Via Antonio di Rudinì, 8, 20142 Milan, Italy; 2 Young Otolaryngologists-International Federation of Otorhinolaryngological Societies (Yo-IFOS), Paris, France; 3 Maxillofacial Surgery Unit, ASST Santi Paolo E Carlo, Department of Health Sciences, Università Degli Studi Di Milano, Milan, Italy; 4 Division of Otolaryngology-Head and Neck Surgery, Centre Hospitalier de L’Université de Montréal, Montreal, QC Canada; 5 Department of Otorhinolaryngology and Head and Neck Surgery, University Clinical Hospital Centre Sestre Milosrdnice, Zagreb University School of Medicine, Zagreb, Croatia; 6 Confederation of European Otorhinolaryngology-Head and Neck Surgery (CEORL-HNS), Vienna, Austria; 7 Rhinology and Skull Base Unit, Department of Otorhinolaryngology, Hospital Clinic, University of Barcelona, Barcelona, Catalonia Spain; 8 Richard J McNally Laboratory, Harvard University, Cambridge, MA USA; 9 Freeman Hospital, Newcastle, UK; 10 Department of Oto-Rhino-Laryngology, Head and Neck Surgery, and Audiology, Copenhagen University Hospital Rigshospitalet, Copenhagen, Denmark; 11 Private Practice, Ganshoren, Belgium; 12 Department of Infectious Diseases, CHU Saint-Pierre, Université Libre de Bruxelles (ULB), Brussels, Belgium; 13 School of Public Health, Université Libre de Bruxelles (ULB), Brussels, Belgium; 14 Institute for Medical Immunology, Brussels, Belgium; 15 Department of ORL-HNS, University of Limerick Medical School, Limerick, Ireland; 16 Department of Head and Neck Surgery, Poznan University of Medical Sciences, The Greater Poland Cancer Centre, Poznan, Poland; 17 Center for Rhinology and Allergology, Wiesbaden, Germany; 18 Division of Otolaryngology and Head and Neck Surgery, University of Montréal, Montreal, Canada; 19 Department of Medicine, Jewish General Hospital, Montreal, Canada; 20 Head and Neck Oncology and Robotic Surgery, Azienda Ospedaliero Universitaria Careggi, Firenze, Italy; 21 Otorhinolaryngology-Head and Neck Surgery Department, Complexo Hospitalario Universitario A Coruña (CHUAC), A Coruña, Galicia Spain; 22 Clinical Research in Medicine, International Center for Doctorate and Advanced Studies (CIEDUS), Universidade de Santiago de Compostela (USC), Santiago de Compostela, Galicia Spain; 23 Department of ORL-HNS, Ankara University Medical School, Ankara, Turkey; 24 Department of ORL-HNS, Salzburg Paracelsus Medical University, Salzburg, Austria; 25 Kasr Alainy School of Medicine, Cairo University, Giza, Egypt; 26 Department of Otorhinolaryngology and Head and Neck Surgery, CHU de Lille, Université de Lille 2, Lille, France; 27 Department of Otorhinolaryngology, Head and Neck Surgery, Medical University of Warsaw, Warsaw, Poland; 28 University Clinic of ORL-HNS, Aarhus University Hospital, Aarhus, Denmark; 29 Department of Clinical Science, Intervention and Technology (CLINTEC), Karolinska Institutet, Stockholm, Sweden; 30 Department of Otorhinolaryngology and Head and Neck Surgery, 1st Faculty of Medicine, Charles University, Motol University Hospital, Prague, Czech Republic; 31 Department of ORL-Head & Neck Surgery, Centre Hospitalier du Luxembourg, Luxembourg, Luxembourg; 32 Department of Gastroenterology, Neurosciences, Experimental ORL, Neurogastroenterology & Motility, Deglutology-University Hospitals Leuven, University of Leuven, Leuven, Belgium; 33 Department of Otolaryngology, Charing Cross and Royal Brompton Hospitals, Imperial College, London, UK; 34 Department of Neonatology, Poznan University of Medical Sciences, Poznan, Poland; 35 Medical Faculty, Comenius University, Bratislava, Slovakia; 36 Infectious Disease Unit, ASST Santi Paolo E Carlo, Department of Health Sciences, Università Degli Studi Di Milano, Milan, Italy; 37 Department of Otorhinolaryngology and Head and Neck Surgery, Jerez University Hospital, Jerez de la Frontera, Spain; 38 Department of Otorhinolaryngology and Head and Neck Surgery, University Hospital Ostrava and Faculty of Medicine, University of Ostrava, Ostrava, Czech Republic; 39 Department of Otolaryngology-Head and Neck Surgery, Foch Hospital, Paris Saclay University, Saint-Aubin, France

**Keywords:** Coronavirus infections, Health planning guidelines, Vaccine, Healthcare workers, Pregnancy, Breastfeeding, Covid-19

## Abstract

**Purpose:**

SARS-CoV-2 vaccines are a key step in fighting the pandemic. Nevertheless, their rapid development did not allow for testing among specific population subgroups such as pregnant and breastfeeding women, or elaborating specific guidelines for healthcare personnel working in high infection risk specialties, such as otolaryngology (ORL). This clinical consensus statement (CCS) aims to offer guidance for SARS-CoV-2 vaccination to this high-risk population based on the best evidence available.

**Methods:**

A multidisciplinary international panel of 33 specialists judged statements through a two-round modified Delphi method survey. Statements were designed to encompass the following topics: risk of SARS-Cov-2 infection and use of protective equipment in ORL; SARS-Cov-2 infection and vaccines and respective risks for the mother/child dyad; and counseling for SARS-CoV-2 vaccination in pregnant, breastfeeding, or fertile healthcare workers (PBFHW). All ORL PBFHW were considered as the target audience.

**Results:**

Of the 13 statements, 7 reached consensus or strong consensus, 2 reached no consensus, and 2 reached near-consensus. According to the statements with strong consensus otorhinolaryngologists—head and neck surgeons who are pregnant, breastfeeding, or with childbearing potential should have the opportunity to receive SARS-Cov-2 vaccination. Moreover, personal protective equipment (PPE) should still be used even after the vaccination.

**Conclusion:**

Until prospective evaluations on these topics are available, ORL-HNS must be considered a high infection risk specialty. While the use of PPE remains pivotal, ORL PBFHW should be allowed access to SARS-CoV-2 vaccination provided they receive up-to-date information.

**Supplementary Information:**

The online version contains supplementary material available at 10.1007/s00405-021-06794-6.

## Introduction

The coronavirus disease 2019 (COVID-19) is one of the most important worldwide pandemics of the last decades [1].

Many clinical studies reported that a large number of physicians had to face increased infectious risk and contamination during the delivery care process [2, 3]. Among them, otolaryngologists—head and neck surgeons (ORL-HNS) were particularly at risk, given the tropism of the severe acute respiratory syndrome coronavirus 2 (SARS-CoV-2) for the nasopharyngeal epithelium, and the high number of daily practice procedures involving nasal or oral cavities [4]. Higher viral loads are found in the nose as compared to the throat suggesting a higher risk of contamination when working in this specific area [5]. This risk is increased in case of emergency procedures such as management of major epistaxis or airway obstruction. In these situations, the practitioner does not have time to perform and wait for the result of a nasal swab [6].

The development of two mRNA vaccines for SARS-CoV-2 appeared to be a key step in the abating process of the pandemic. Furthermore, the first two vaccines were rapidly joined also by an adenoviral vector vaccine and a recombinant vaccine. Nevertheless, the rapid development of these vaccines raised safety concerns not only from the public [7] but also from healthcare providers [8]. Moreover, it did not allow for extensive testing among specific population subgroups such as pregnant and breastfeeding women, despite a massive call for action for experimentation also in these patients, differently from what happened for other vaccines in the past [9]. Consequently, the expected response to the vaccination campaign is extremely variable and unique recommendations for risk categories, most notably pregnant women [7], are still lacking. Accumulated evidence demonstrated pregnancy is a risk factor for developing a severe form of COVID-19 [10]. Moreover, SARS-CoV-2 infection seems to be associated with a higher risk of preterm delivery [11].

In that way, some experts from the Society for Maternal–Fetal Medicine Health Policy Advocacy Committee recently supported that the vaccination of pregnant, breastfeeding, and fertile health workers (PBFHW) makes particularly sense (see Online Resource 1) [12].

The aim of this clinical consensus statement paper (CCS) is to offer, through a modified Delphi process, specific guidance, and advice based on the best evidence presently available.

## Methods

The development of this CCS followed the modified Delphi protocol proposed by Rosenfeld et al. [13] which is based on the following steps: (1) evaluating whether vaccines for SARS-CoV-2 in OTO-HNS PBFHW is an appropriate topic for a CCS, (2) determining the scope and population of interest, (3) expert panel recruitment, (4) vetting panelists’ potential conflict of interests, (5) performing a systematic literature review, (6) performing modified Delphi surveys, (7) iteratively revising clinical statements, and (8) aggregating the data. Due to the nature of the study, no specific approval by an Internal Review Board was required.

### Panelists and scope of consensus statement

The panel was composed of 33 collaborators from 18 countries (see Online Resource 2 for a map of contributions to the CCS). The development group consisted of a chair (AMS), assistant chair (JL), and methodologist (FA). ORL-HNS authors were recruited regarding their expertise among the Young Otolaryngologist—International Federation of Otorhinolaryngological Societies (YO-IFOS) research group and the Confederation of European Otorhinolaryngology—Head and Neck Surgery (CEORL-HNS) board. The panel was composed of 27 ORL-HNS, one epidemiologist, two infectious disease specialists, one vaccinologist, one gynecologist, and one neonatologist. All of them are working in the field of COVID-19. A single case of conflict of interest emerged among the authors, which was deemed as not relevant to the paper by the development committee. The focus of the CCS was to offer specific guidance to ORL-HNS PBFHW for SARS-CoV-2 vaccination.

### Literature review

A PRISMA-compliant systematic review of the literature was conducted around four topics: (1) vaccines for SARS-CoV-2 for ORL-HNS PBFHW; (2) vaccines for SARS-CoV-2 for all PBFHW; (3) risk and prevention of SARS-CoV-2 infection in ORL-HNS; and (4) SARS-CoV-2 infection in PBFHW. MEDLINE, EMBASE, and Web of Science databases were searched on December, 28th for studies in English, Italian, German, French, or Spanish that reported data obtained from human subjects. Example search keys are shown in Online Resource 3. Searches for topic 1 delivered no results. Due to a lack of high-quality studies on topics 2 and 3, the systematic review was extended from what has been recommended for CCSs [13] to include all studies published on the topics.

Upon evaluating the literature recovered through the systematic review, a collection of 84 articles, representing the highest and most recent evidence on the topics, was prepared and distributed to all authors to be revised over 1 week (see Online Resource 4 for the full references of the distributed literature).

#### Clinical statement development and modified Delphi survey

Based on the literature review and the aim of the CCS, the chair and assistant chair developed the core clinical statements for the survey, which were further discussed and expanded by the ORL-HNS group, and finally edited by the methodologist.

Statements were developed based on the literature review and the development group’s perception of important clinical scenarios. A final 13-statement survey was, therefore, created and distributed to authors using Google Forms (Google LLC, Mountain View, CA, US). Authors were instructed to complete the survey anonymously. Authors were asked to report their agreement with each statement according to a nine-point Likert scale from strongly disagree (1) to strongly agree (9). As defined by Rosenfeld [13], the results for each statement were defined as follows: strong consensus: mean score of ≥ 8.00 with no outliers (defined as any rating 2 or more Likert points from the mean in either direction); consensus: mean score of ≥ 7.00 with no more than 1 outlier; near-consensus: mean score of ≥ 6.50 with no more than 2 outliers; and no consensus: all other statements.

After the first survey round, 4 of 13 statements reached a consensus, and 9 reached no consensus. No statement reached near-consensus. The seven no consensus items with a mean score > 6.5 were reworded based on anonymous comments from authors. While the ultimate content of each statement was not changed, the statements were improved formally in terms of clarity. The second survey round included seven statements, of which three reached a consensus, two reached near-consensus, and two did not reach consensus. As there were not enough comments to guide the third Delphi round and timely publication of results was preferred, the Delphi round was closed with the second round.

## Results

The first Delphi round was completed by all panelists, while the second round was completed by 30 out of 33 panelists. After the two Delphi rounds, 7 out of 13 statements reached a consensus, two reached near-consensus, and 4 did not reach consensus. The evolution of statements from the first round to their final version is reported in Online Resource 5. Delphi process results for all statements along with their mean score, score range, and the respective number of outliers are reported in Table 1.

**Table 1:** Statements and results from the Delphi process

Question number	Statement	Mean	Range	Outliers	Delphi round
*ODS diagnosis statements that reached consensus*					
1	Otolaryngology and head and neck surgery represent specialties at high risk of SARS-CoV2 infection	8.47	1–9	1	1
2	Although preventive measures and use of full personal protective equipment has been demonstrated to prevent SARS-CoV2 infection, due to environmental, behavioral, and practical contingencies, the specialty-related risk of infection can be minimized but not completely removed	8.4	6–9	1	2
4	Though the recently developed SARS-CoV2 mRNA vaccines do not seem to show a risk profile for complication for the mother–baby dyad during pregnancy and breastfeeding, we have no experimental data in this population on which no trial has been conducted and no long-term evaluation is available	7.97	5–9	1	2
5	All pregnant, breastfeeding, or fertile female otolaryngologists and head and neck surgeons considering a COVID-19 vaccine should have access to up-to-date information about the safety and efficacy of the vaccine for the mother–baby dyad, including clear information about data and evidence that are not available yet for this specific population	8.47	1–9	1	1
6	All pregnant otolaryngologists and head and neck surgeons who are active in clinical practice should be given the opportunity to receive the SARS-CoV2 vaccine rapidly, provided the choice is free, individual, and informed and assisted by a health professional to individually assess the benefits and risks according to each case	8.47	7–9	0	2
11	All pregnant and breastfeeding otolaryngologists and head and neck surgeons who decline vaccination should be strongly stimulated to keep in mind prevention measures such as hand washing, physical distancing, wearing a mask, and using proper personal protection devices	8.72	5–9	1	1
12	The use of adequate personal protective equipment against SARS-CoV2 remains strongly recommended for otolaryngologist and head and neck surgeons who received the SARS-CoV2 vaccine	8.63	7–9	0	1
*Statements that reached near-consensus*					
8	All breastfeeding otolaryngologists and head and neck surgeons should be given the opportunity to receive the SARS-CoV2 vaccine, provided the choice is free, individual, and informed and assisted by a health professional to individually assess the benefits and risks according to each case	8.17	5–9	2	2
13	Since prenatal maternal stress is also associated with neurodevelopmental disorders among exposed offspring, all pregnant otolaryngologists and head and neck surgeons should take into account in the informed and assisted decision to take the SARS-Cov2 vaccine not only the infection risk but also the psychological burden imposed by the risk of SARS-CoV2 infection, adequately balanced to that of receiving the SARS-CoV2 vaccine	7.67	1–9	2	2
*Statements that did not reach consensus*					
3	Pregnant people with COVID-19 might be at increased risk of adverse pregnancy outcomes compared with pregnant women without COVID-19 and, although chances for severe health effects are low, pregnant people with COVID-19 have an increased risk of severe illness compared with non-pregnant women of reproductive age	7.73	5–9	3	2
7	All pregnant otolaryngologists and head and neck surgeons who are active in clinical practice may be encouraged to receive the SARS-CoV2 vaccine rapidly, provided the choice is free, individual, and informed and assisted by a health professional to individually assess the benefits and risks according to each case	7.5	3–9	4	2
9	All breastfeeding otolaryngologists and head and neck surgeons who are not active in clinical practice and do not expect to resume clinical practice before stopping breastfeeding, should wait for the end of breastfeeding before receiving the SARS-CoV2 vaccine and use appropriate contraception prior to vaccination and up to 2 months after receiving the second vaccine dose	6.41	3–9	4	1
10	All non-pregnant and non-breastfeeding otolaryngologists and head and neck surgeons of childbearing potential who opt for receiving the SARS-CoV2 vaccine should use appropriate contraception prior to vaccination and up to 2 months after receiving the second vaccine dose	6.22	3–9	6	1

Two statements reached a strong consensus: statement 6 (on the opportunity for receiving vaccines for pregnant ORL-HNS), and statement 12 (suggesting ORL-HNS maintain the use of PPE even after the vaccination).

Also, all near-consensus scoring rated > 7.5 on average, mirroring a general trend towards consensus in the context of few variable opinions.

## Discussion

A multidisciplinary group of experts involved in research on the COVID-19 pandemic and with significant experience in the management of the disease contributed to this CCS. There was a positive general attitude towards the opportunity for vaccines for SARS-CoV-2 vaccination in ORL-HNS PBFHW. Nevertheless, the response from the panel mirrored a mixed attitude. In the present paper, a consensus was reached for more than half of the statements. This represents a more than satisfactory result, given the strict criteria required by the modified Delphi method as proposed by Rosenfeld et al. [13].

As no study has yet to be able to delve into the role of SARS-CoV-2 vaccines for ORL-HNS specialists, this CCS fills out a gap in the current literature on the subject. The main result of this CCS is recognizing the risk of infection for ORL-HNS specialists and the importance of vaccination for pregnant colleagues. Moreover, the opportunity for vaccination for breastfeeding ORL-HNS reached only a near-consensus, albeit with an extremely high score. Last, the panel felt as evidence was not sufficient to build a consensus towards colleagues with childbearing potential. Nevertheless, it is worth reiterating that, above anything else, adhering to the SARS-CoV-2 vaccination campaign should always represent an option and not an obligation for any pregnant, breastfeeding, or childbearing age woman. On the other hand, having access to information to make this free choice also a conscious choice is mandatory. This aspect is highlighted when comparing the results of statements 6 and 7 (“all pregnant otolaryngologists and head and neck surgeons […] should be given the opportunity to receive the SARS-CoV2 vaccine rapidly” vs. “All pregnant otolaryngologists and head and neck surgeons […] may be encouraged to receive the SARS-CoV2 vaccine rapidly”). While allowing the opportunity for vaccination reached a strong consensus, the encouragement for vaccination failed to reach any consensus among experts.

This CCS reflects the simple, yet not predictable, rationale briefly presented in the introduction. Such reasoning is based on the data collected from the available literature in the systematic review. Such data will be briefly covered in this article to maximize its informative content.

1. In the context of a high rate of SARS-CoV-2 infection in healthcare workers [14], ORL-HNS have a higher risk of SARS-CoV-2 infection [15, 16]. Such risk can be reduced by awareness [17], prevention measures, and the adequate use of personal protective equipment [18–20]. This is demonstrated by the high price paid by these specialists in terms of infections and victims during the pandemic [21]. It has to be noted though that despite recommendations for introduction of routine testing of patients for SARS-CoV-2, not all emergency or outpatient clinic settings allow for it [22]. Last, we are not able to postpone elective surgeries ad libitum as initially proposed [23–25]. Somehow, ORL-HNS staff must be trained to better respond to potential infection and leaves among colleagues [26]. In that way, the vaccine may represent a powerful tool for ensuring that our specialties may meet the demands of the general population. This general framework was positively accepted by the whole CCS, as mirrored by scores of statements 1, 2, 11, and 12.
2. SARS-CoV-2 represents a risk for pregnancy with indirect negative effects on the newborns [27]. SARS-CoV-2 infection during pregnancy has been diffusely associated with preterm delivery, with fetal growth restriction and with more severe forms of COVID-19 illness for mothers [11]. Consequently, a non-negligible percentage of newborns required Intensive Care Unit Admission [28, 29]. Furthermore, the infection risk was associated with a heavier psychological burden for mothers [30–32]. SARS-CoV-2 vertical transmission is infrequent but is ascertained in several cases. Furthermore, the presence of a viral load in the placenta and milk has been demonstrated [28]. This latter feature though has not been linked to horizontal infective risk, as the secreted virus is being considered an infection incompetent [33]. Breastfeeding is, therefore, recommended even in case of maternal infection. This has been shown as one of the few points of convergence of pregnancy management guidelines worldwide [34, 35]. Mask use and careful disinfection of hand and breast remain a staple of babies’ protection from environmental infection during breastfeeding. The separation between mother and newborn is usually not recommended [34]. Nevertheless, the evidence gathered nevertheless felt not enough univocal and strong. Consequently, we failed to reach a consensus on the related items (3 and 13, the latter though reaching near-consensus with a good score). For what concerns item 3, which addresses a pivotal aspect of the relationship between COVID-19 and motherhood, the three remaining outliers from the second Delphi round scored the statement 5. Two outliers did not support their choice with any comment. The third panelist stated that pregnancy could act as a kind of protection against COVID-19. It has to be noted that this hypothesis was consistent with early reports from China [36], reports which were later denied by data from larger cohorts.
3. The vaccines for SARS-CoV-2 may not represent a risk for the mother–baby dyad during pregnancy and breastfeeding. No specific trials have been conducted yet in these two populations, despite a sound request by the scientific community [9, 37, 38]. Nevertheless, the characteristics of this new mRNA vaccine appear safe in pregnant and breastfeeding women from a biological perspective [39, 40]. mRNA vaccines are indeed not live virus vaccines, nor do they use an adjuvant to enhance vaccine efficacy. These vaccines do not enter the nucleus and do not alter DNA in vaccine recipients, as demonstrated in animal models [39, 41]. In that way, it seems impossible that mRNA vaccines might cause any genetic changes, as mRNA lasts only 24–36 h in the cell before degradation. These assumptions led several agencies and societies to suggest offering the vaccines for SARS-CoV-2 to pregnant and breastfeeding women [42–45]. This position has been supported not only from a merely scientific standpoint but also from an ethical perspective, as reported by the PREVENT working group [46]. It has to be noted that every recommendation for SARS-CoV-2 vaccination during pregnancy suggests considering the risk of viral infection and complications against the unassessed safety profile of the vaccine. Furthermore, among the 15 pregnancy cases who received approved vaccines for SARS-CoV-2 [47, 48] there were no complications. The general lack of evidence and the novelty of the vaccines turned into the most diverse response from the panel. The inadequacy of scientific evidence on the vaccine and the need for informing PBFHW on risks and benefits were recognized by the CCS (statements 4 and 5). The scientific context was deemed adequate to support the opportunity for pregnant ORL-HNS vaccination with strong consensus but not sufficient to fully support the opportunity for breastfeeding ORL-HNS vaccination. Unfortunately, the motivation for the near-consensus about statement 8 (which had two outliers scoring 5), were not disclosed by the panelists. Furthermore, the panel judged presently available information not adequate to support postponing the vaccination in inactive breastfeeding ORL-HNS. Analogously, they did not suggest temporary contraception after vaccination in non-pregnant and non-breastfeeding colleagues with childbearing potential.

This CCS acquires a specific role if we frame it in the complex picture of public reaction to vaccines for SARS-CoV-2, a topic that has shifted from scientific debate to media sensation no differently than for any news related to COVID-19, from infection rates to containment measures and potential therapies. Despite that the vaccines for SARS-CoV-2 development represent a long-awaited scientific breakthrough, the expected response rate to the call to vaccination is variable, even among healthcare professionals [7, 8]. This is not only due to a rising serpentine diffidence towards vaccines, unbacked by scientific data [49], but also to the technical novelty of these vaccines, the first one being based on the inoculation of synthetized viral mRNA.

Still, there are other aspects to take into account when evaluating the relationship between SARS-CoV-2 infection and human fertility. Such topics have not been covered in this CCS as they concern the vaccine issue only marginally and there is little pertinent scientific information is available. First, we currently do not have any conclusive data on the role of SARS-Cov-2 infection and human fertility. Another important issue is the unexplored role of this pathogen in maternal immunization. Beyond maternal protection, maternal immunization against SARS-CoV-2 might contribute to the protection of the neonate in the postnatal period, as already demonstrated with other vaccines such as pertussis [50]. Lastly, we have no data on the duration of the immunization related to vaccines and on the risk of asymptomatic infection and shedding risk [51]. Therefore, the use of personal protection devices remains strongly recommended for all healthcare workers.

Last, it is helpful to recall that this study, albeit structured with the utmost scientific rigor and based on all available evidence, still represents an opinion paper on a rapidly evolving topic. While we confirm our commitment to providing information and guidance to all ORL-HNS PBFHW, we cannot underestimate the role of future prospective evaluations on these topics which we hope will be soon available for scrutiny from the scientific community.

## Supplementary Information

Below is the link to the electronic supplementary material.

Supplementary file1 (PDF 207 KB)

Supplementary file2 (PDF 290 KB)

Supplementary file3 (PDF 256 KB)

Supplementary file4 (PDF 313 KB)

Supplementary file5 (PDF 248 KB)

## Abbreviations

ORL-HNS: Otolaryngology and head and neck surgery
PBFHW: Pregnant, breastfeeding, and fertile health workers
CCS: Clinical consensus statement
