# Supplementary material for: Appropriateness for SARS-CoV-2 vaccination for otolaryngologist and head and neck surgeons in case of pregnancy, breastfeeding, or childbearing potential: Yo-IFOS and CEORL-HNS joint clinical consensus statement
Source: Eur Arch Otorhinolaryngol. 2021 Apr 15;278(10):4091–9. doi: 10.1007/s00405-021-06794-6 (PMC8046580; doi:10.1007/s00405-021-06794-6)
Supplement: Supplementary file 1 — Supplementary file1 (PDF 207 KB) [file 405_2021_6794_MOESM1_ESM.pdf]

**Appropriateness for SARS-CoV-2 Vaccination for Otolaryngologist and Head and Neck Surgeons in case of Pregnancy, Breastfeeding or Childbearing potential: Yo-IFOS and CEORL-HNS joint clinical consensus statement**

**Journal: European Archives of Oto-Rhino-Laryngology**

Authors: Saibene Alberto Maria, et al.

Correspondence to: Alberto Maria Saibene, Otolaryngology Unit - ASST Santi Paolo e Carlo. Via Antonio di Rudinì, 8 - 20142 - Milan, Italy. Phone: +39 02 8184 4249. Fax: +39 02 5032 3166. Mail: alberto.saibene@gmail.com

**Online resource 1: Reasons supporting healthcare workers vaccination from the Society for Maternal-Fetal Medicine Health Policy Advocacy Committee (from Maykin MM, Heuser C, Feltovich H, with the Society for Maternal-Fetal Medicine Health Policy Advocacy Committee. Pregnant people deserve the protection offered by SARS-CoV-2 vaccines. Vaccine. 2020. doi:10.1016/j.vaccine.2020.12.007)**

| |
|---|
| (a) outcomes in pregnancy are equivalent or worse than in non-pregnant populations, |
| (b) there is potential for harm to not one but two lives, |
| (c) persons of childbearing potential may have increased workplace exposure to SARS-CoV-2,and |
| (d) inadvertent vaccination of pregnant persons is inevitable |
