# Supplementary material for: Appropriateness for SARS-CoV-2 vaccination for otolaryngologist and head and neck surgeons in case of pregnancy, breastfeeding, or childbearing potential: Yo-IFOS and CEORL-HNS joint clinical consensus statement
Source: Eur Arch Otorhinolaryngol. 2021 Apr 15;278(10):4091–9. doi: 10.1007/s00405-021-06794-6 (PMC8046580; doi:10.1007/s00405-021-06794-6)
Supplement: Supplementary file 2 — Supplementary file2 (PDF 290 KB) [file 405_2021_6794_MOESM2_ESM.pdf]

Appropriateness for SARS-CoV-2 Vaccination for Otolaryngologist and Head and Neck Surgeons in case of Pregnancy, Breastfeeding or Childbearing potential: YO-IFOS and CEORL-HNS joint clinical consensus statement

Journal: European Archives of Oto-Rhino-Laryngology

Authors: Saibene Alberto Maria, et al.

Correspondence to: Alberto Maria Saibene, Otolaryngology Unit - ASST Santi Paolo e Carlo. Via Antonio di Rudinì, 8 - 20142 - Milan, Italy. Phone: +39 02 8184 4249. Fax: +39 02 5032 3166. Mail: alberto.saibene@gmail.com

Online resource 2: Map of contributions to the clinical consensus statement. States where a panelist from the clinical consensus statement worked while building the consensus are colored in green

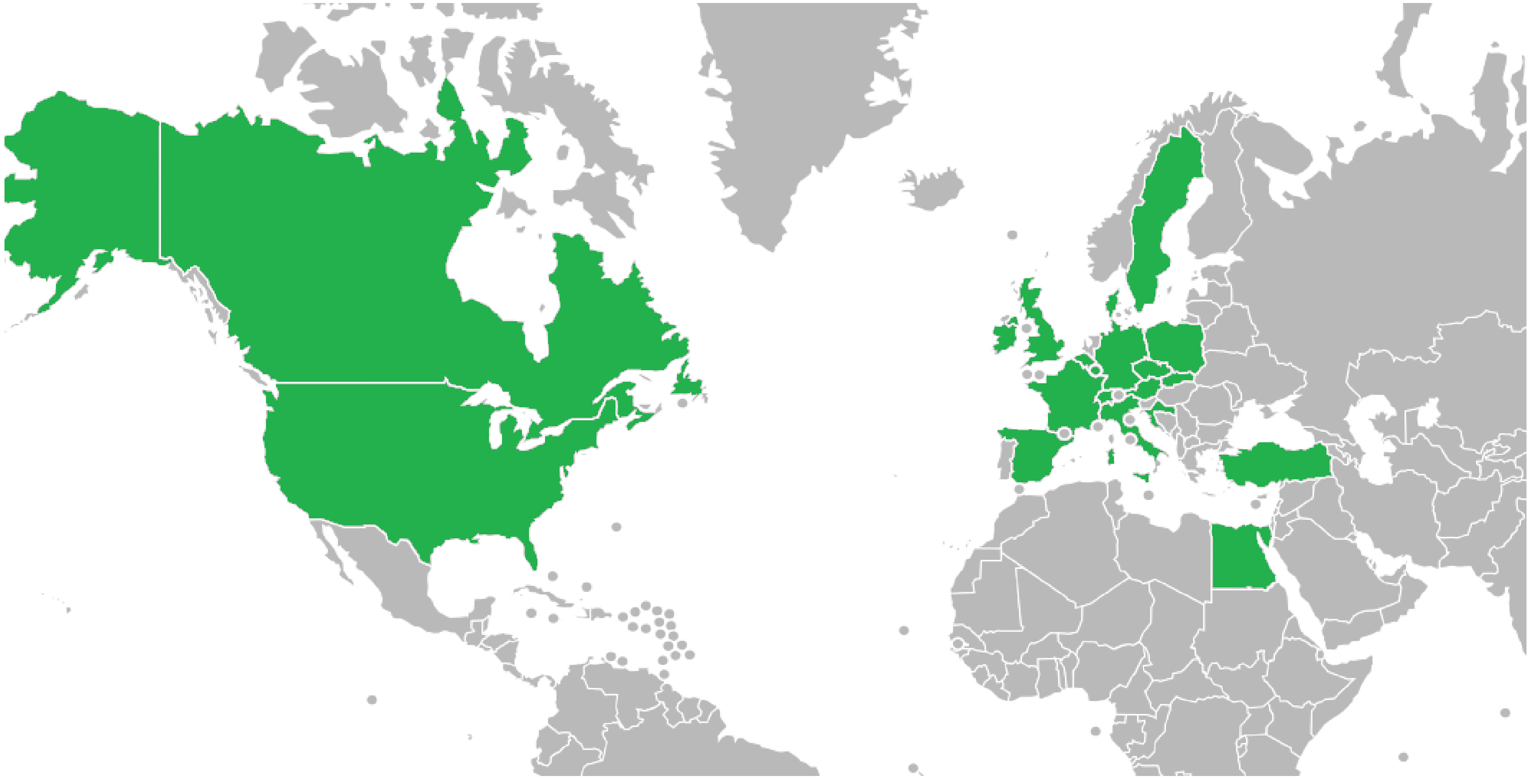

(full, original, uncolored vector image courtesy of Geordie Bosanko, distributed under Creative Commons license and available at https://commons.wikimedia.org/wiki/File:Mercator_Projection.svg)
