# Supplementary material for: Appropriateness for SARS-CoV-2 vaccination for otolaryngologist and head and neck surgeons in case of pregnancy, breastfeeding, or childbearing potential: Yo-IFOS and CEORL-HNS joint clinical consensus statement
Source: Eur Arch Otorhinolaryngol. 2021 Apr 15;278(10):4091–9. doi: 10.1007/s00405-021-06794-6 (PMC8046580; doi:10.1007/s00405-021-06794-6)
Supplement: Supplementary file 3 — Supplementary file3 (PDF 256 KB) [file 405_2021_6794_MOESM3_ESM.pdf]

Appropriateness for SARS-CoV-2 Vaccination for Otolaryngologist and Head and Neck Surgeons in case of Pregnancy, Breastfeeding or Childbearing potential: Yo-IFOS and CEORL-HNS joint clinical consensus statement

Journal: European Archives of Oto-Rhino-Laryngology

Authors: Saibene Alberto Maria, et al.

Correspondence to: Alberto Maria Saibene, Otolaryngology Unit - ASST Santi Paolo e Carlo. Via Antonio di Rudinì, 8 - 20142 - Milan, Italy. Phone: +39 02 8184 4249. Fax: +39 02 5032 3166. Mail: alberto.saibene@gmail.com

Online resource 3: Search strategy for MEDLINE database for all consulted topics

**Topic**	**Search strategy**	**Date of search**	**Unique items found**
Covid-19 vaccine during Pregnancy or Breastfeeding and in women of Childbearing potential for Otolaryngologist and Head and Neck Surgeons	((("sars-cov-2" OR "COVID-19" OR "new coronavirus 2019") AND (vaccine OR vaccination)) AND (pregnancy OR breastfeeding OR childbearing)) AND (otolaryngologist OR "ENT surgeon" OR "ENT specialist" OR otorhinolaryngologist OR "neck surgeon"OR otolaryngology OR otorhinolaryngology OR "neck surgery")	December, the 28th, 2020	0
Covid-19 vaccine during Pregnancy or Breastfeeding and in women of Childbearing potential	(("sars-cov-2" OR "COVID-19" OR "new coronavirus 2019") AND (vaccine OR vaccination)) AND (pregnancy OR breastfeeding OR childbearing)		53
Risk and prevention of SARS-CoV-2 infection in Otolaryngologist and Head and Neck Surgeons *[search limited to title and abstract]*	(("sars-cov-2"[Title/Abstract] OR "COVID-19"[Title/Abstract] OR "new coronavirus 2019")[Title/Abstract] AND (otolaryngologist[Title/Abstract] OR "ENT surgeon"[Title/Abstract] OR "ENT specialist"[Title/Abstract] OR otorhinolaryngologist[Title/Abstract] OR "head AND neck surgeon"[Title/Abstract] OR otolaryngology[Title/Abstract] OR otorhinolaryngology[Title/Abstract] OR "headAND neck surgery"))[Title/Abstract] AND (exposure[Title/Abstract] OR infection[Title/Abstract] OR "infection risk"[Title/Abstract] OR transmission)[Title/Abstract]		184
SARS-Cov-2 infection in case of pregnancy, breastfeeding, and childbearing potential *[search limited to title and abstract, ant to guidelines and systematic reviews]*	(("sars-cov-2"[Title/Abstract] OR "COVID-19"[Title/Abstract] OR "new coronavirus 2019"[Title/Abstract]) AND (guideline[Title/Abstract] OR guidelines[Title/Abstract] OR "systematic review"[Title/Abstract])) AND (breastfeeding[Title/Abstract] OR pregnancy[Title/Abstract] OR "childbearing potential"[Title/Abstract])		178
