# Supplementary material for: Appropriateness for SARS-CoV-2 vaccination for otolaryngologist and head and neck surgeons in case of pregnancy, breastfeeding, or childbearing potential: Yo-IFOS and CEORL-HNS joint clinical consensus statement
Source: Eur Arch Otorhinolaryngol. 2021 Apr 15;278(10):4091–9. doi: 10.1007/s00405-021-06794-6 (PMC8046580; doi:10.1007/s00405-021-06794-6)
Supplement: Supplementary file 4 — Supplementary file4 (PDF 313 KB) [file 405_2021_6794_MOESM4_ESM.pdf]

Appropriateness for SARS-CoV-2 Vaccination for Otolaryngologist and Head and Neck Surgeons in case of Pregnancy, Breastfeeding or Childbearing potential: Yo-IFOS and CEORL-HNS joint clinical consensus statement

Journal: European Archives of Oto-Rhino-Laryngology

Authors: Saibene Alberto Maria, et al.

Correspondence to: Alberto Maria Saibene, Otolaryngology Unit - ASST Santi Paolo e Carlo. Via Antonio di Rudinì, 8 - 20142 - Milan, Italy. Phone: +39 02 8184 4249. Fax: +39 02 5032 3166. Mail: alberto.saibene@gmail.com

Online resource 4: References for the literature distributed among consensus panelists, according to topic

1. Covid-19 vaccine during Pregnancy or Breastfeeding and in women of Childbearing potential

1. COVID-19 vaccines. Drugs and Lactation Database (LactMed) [Internet]. National Library of Medicine (US); 2020.

2. Mehrabani S. COVID-19 Infection and Children: A Comprehensive Review. Int J Prev Med. 2020;11: 157.

3. Maykin MM, Heuser C, Feltovich H, with the Society for Maternal-Fetal Medicine Health Policy Advocacy Committee. Pregnant people deserve the protection offered by SARS-CoV-2 vaccines. Vaccine. 2020. doi:10.1016/j.vaccine.2020.12.007

4. Madjunkov M, Dviri M, Librach C. A comprehensive review of the impact of COVID-19 on human reproductive biology, assisted reproduction care and pregnancy: a Canadian perspective. J Ovarian Res. 2020;13: 140.

5. Mascio DD, Di Mascio D, Sen C, Saccone G, Galindo A, Grünebaum A, et al. Risk factors associated with adverse fetal outcomes in pregnancies affected by Coronavirus disease 2019 (COVID-19): a secondary analysis of the WAPM study on COVID-19. Journal of Perinatal Medicine. 2021. pp. 111–115. doi:10.1515/jpm-2020-0539

6. Juan J, Gil MM, Rong Z, Zhang Y, Yang H, Poon LC. Effect of coronavirus disease 2019 (COVID-19) on maternal, perinatal and neonatal outcome: systematic review. Ultrasound Obstet Gynecol. 2020;56: 15–27.

7. Jaffe E, Lyerly AD, Goldfarb IT. Pregnant women's perceptions of risks and benefits when considering participation in vaccine trials. Vaccine. 2020. pp. 6922–6929. doi:10.1016/j.vaccine.2020.08.059

8. Dashraath P, Nielsen-Saines K, Madhi SA, Baud D. COVID-19 vaccines and neglected pregnancy. Lancet. 2020;396: e22.

9. Khalil A, Kalafat E, Benlioglu C, O'Brien P, Morris E, Draycott T, et al. SARS-CoV-2 infection in pregnancy: A systematic review and meta-analysis of clinical features and pregnancy outcomes. EClinicalMedicine. 2020;25: 100446.

10. Whitehead CL, Walker SP. Consider pregnancy in COVID-19 therapeutic drug and vaccine trials. The Lancet. 2020. p. e92. doi:10.1016/s0140-6736(20)31029-1

2. Risk and prevention of SARS-CoV-2 infection in Otolaryngologist and Head and Neck Surgeons

11. Bleier BS, Ramanathan M, Lane AP. COVID-19 Vaccines May Not Prevent Nasal SARS-CoV-2 Infection and Asymptomatic Transmission. Otolaryngology–Head and Neck Surgery. 2020. p. 019459982098263. doi:10.1177/0194599820982633

12. Chan Y, Banglawala SM, Chin CJ, Côté DWJ, Dalgorf D, de Almeida JR, et al. CSO (Canadian Society of Otolaryngology - Head & Neck Surgery) position paper on rhinologic and skull base surgery during the COVID-19 pandemic. J Otolaryngol Head Neck Surg. 2020;49: 81.

13. Parilli-Troconis D, Baptista P, Marcano-Lozada M, Goncalves S, Shahal D, Chiossone-Kerdel JA. COVID-19 Infection and Its Influence in Otorhinolaryngology-Head and Neck Surgery. Int Arch Otorhinolaryngol. 2020;24: e527–e534.

14. Chan Y, Angel D, Aron M, Hartl T, Moubayed SP, Smith KA, et al. CSO (Canadian Society of Otolaryngology - Head & Neck Surgery) position paper on return to Otolaryngology - Head & Neck Surgery Clinic Practice during the COVID-19 pandemic in Canada. J Otolaryngol Head Neck Surg. 2020;49: 76.

15. Hojaij FC, Chinelatto LA, Boog GHP, Kasmirski JA, Lopes JVZ, Medeiros VMB. Head and Neck Practice in the COVID-19 Pandemics Today: A Rapid Systematic Review. Int Arch Otorhinolaryngol. 2020;24: e518–e526.

16. Leitmeyer K, Felton M, Chadha NK. Strategies for restarting Pediatric Otolaryngology outpatient clinics after a pandemic-related shutdown such as from COVID-19. Int J Pediatr Otorhinolaryngol. 2020;139: 110414.

17. Ralli M, Candelori F, Cambria F, Greco A, Angeletti D, Lambiase A, et al. Impact of COVID-19 pandemic on otolaryngology, ophthalmology and dental clinical activity and future perspectives. Eur Rev Med Pharmacol Sci. 2020;24: 9705–9711.

18. Ghulam-Smith M, Choi Y, Edwards H, Levi JR. Unique Challenges for Otolaryngology Patients During the COVID-19 Pandemic. Otolaryngol Head Neck Surg. 2020; 194599820954838.

19. Cai Y, Gulati A, Jiam NT, Wai KC, Shuman EA, Pletcher SD, et al. Evolving Otolaryngology Resident Roles and Concerns at the Peak of the US COVID-19 Pandemic. Head Neck. 2020;42: 3712–3719.

20. Kozin ED, Remenschneider AK, Blevins NH, Jan TA, Quesnel AM, Chari DA, et al. American Neurotology Society, American Otological Society, and American Academy of Otolaryngology - Head and Neck Foundation Guide to Enhance Otologic and Neurotologic Care During the COVID-19 Pandemic. Otol Neurotol. 2020;41: 1163–1174.

21. Pearlman AN, Tabaee A, Sclafani AP, Sulica L, Selesnick SH, Kutler DI, et al. Establishing an Office-Based Framework for Resuming Otolaryngology Care in Academic Practice During the COVID-19 Pandemic. Otolaryngol Head Neck Surg. 2020; 194599820955178.

22. Civantos AM, Bertelli A, Gonçalves A, Getzen E, Chang C, Long Q, et al. Mental health among head and neck surgeons in Brazil during the COVID-19 pandemic: A national study. Am J Otolaryngol. 2020;41: 102694.

23. Xu K, Lu X, Liu Z. Our experiences of resuming services in ENT departments in Wuhan, once a COVID-19 epicenter. Am J Otolaryngol. 2020;41: 102678.

24. Grag K, Shubhanshu K. Effect of Covid-19 in Otorhinolaryngology Practice: A Review. Indian Journal of Otolaryngology and Head & Neck Surgery. 2020. doi:10.1007/s12070-020-02040-3

25. Shakrawal N, Rajan N. The Perils of Covid-19 for Otorhinolaryngologists: An Overview. Indian J Otolaryngol Head Neck Surg. 2020; 1–5.

26. Ren J, Yang X, Xu Z, Lei W, Yang K, Kong Y, et al. Prevention of nosocomial COVID-19 infections in Otorhinolaryngology-Head and Neck Surgery. World J Otorhinolaryngol Head Neck Surg. 2020. doi:10.1016/j.wjorl.2020.06.003

27. Marchioni D, Bisi N, Molteni G, Rubini A. Covid-19 and ENT practice: Our experience: ENT outpatient department, ward and operating room management during the SARS-CoV-2 pandemic. Am J Otolaryngol. 2020;41: 102676.

28. Yang X, Zhang Y, Li S, Chen X. Risk factors for anxiety of otolaryngology healthcare workers in Hubei province fighting coronavirus disease 2019 (COVID-19). Soc Psychiatry Psychiatr Epidemiol. 2020. doi:10.1007/s00127-020-01928-3

29. Ye MJ, Sharma D, Rubel KE, Lebo NL, Burgin SJ, Illing EA, et al. Droplet Exposure Risk to Providers From In-Office Flexible Laryngoscopy: A COVID-19 Simulation. Otolaryngol Head Neck Surg. 2020; 194599820952800.

30. Kowalski LP, Imamura R, Castro Junior G de, Marta GN, Chaves ALF, Matos LL, et al. Effect of the COVID-19 Pandemic on the Activity of Physicians Working in the Areas of Head and Neck Surgery and Otorhinolaryngology. Int Arch Otorhinolaryngol. 2020;24: e258–e266.

31. Sowerby LJ, Stephenson K, Dickie A, Lella FAD, Jefferson N, North H, et al. International registry of otolaryngologist-head and neck surgeons with COVID-19. Int Forum Allergy Rhinol. 2020;10: 1201–1208.

32. Radulesco T, Lechien JR, Sowerby LJ, Saussez S, Chiesa-Estomba C, Sargi Z, et al. Sinus and anterior skull base surgery during the COVID-19 pandemic: systematic review, synthesis and YO-IFOS position. Eur Arch Otorhinolaryngol. 2020. doi:10.1007/s00405-020-06236-9

33. See A, Go LK, Teo CEH, Teo NWY, Toh ST. Adaptations of a Tertiary Otorhinolaryngology Head and Neck Surgery Department in Singapore during the COVID-19 Outbreak. Ann Otol Rhinol Laryngol. 2020; 3489420946779.

34. Kohanski MA, Lo LJ, Waring MS. Review of indoor aerosol generation, transport, and control in the context of COVID-19. Int Forum Allergy Rhinol. 2020;10: 1173–1179.

35. Sharma A, Bhardwaj R. COVID-19 and ENT surgery: a brief review of essential precautions and triage. Eur Arch Otorhinolaryngol. 2020. doi:10.1007/s00405-020-06207-0

36. Miller LE, Shaye DA, Lee LN. Considerations for the otolaryngologist in the era of COVID-19: a review of the literature. Curr Opin Otolaryngol Head Neck Surg. 2020;28: 228–234.

37. Smith JD, Chen MM, Balakrishnan K, Sidell DR, di Stadio A, Schechtman SA, et al. The Difficult Airway and Aerosol-Generating Procedures in COVID-19: Timeless Principles for Uncertain Times. Otolaryngol Head Neck Surg. 2020;163: 934–937.

38. Lagos AE, Ramos PH, Andrade T. Protection for Otolaryngologic Surgery in the COVID-19 Pandemic. OTO Open. 2020;4: 2473974X20934734.

39. Krajewska Wojciechowska J, Krajewski W, Zub K, Zatoński T. Review of practical recommendations for otolaryngologists and head and neck surgeons during the COVID-19 pandemic. Auris Nasus Larynx. 2020;47: 544–558.

40. Mukerji SS, Liu Y-C (carol), Musso MF. Pediatric otolaryngology workflow changes in a community hospital setting to decrease exposure to novel coronavirus. International Journal of Pediatric Otorhinolaryngology. 2020. p. 110169. doi:10.1016/j.ijporl.2020.110169

41. Civantos AM, Byrnes Y, Chang C, Prasad A, Chorath K, Poonia SK, et al. Mental health among otolaryngology resident and attending physicians during the COVID-19 pandemic: National study. Head Neck. 2020;42: 1597–1609.

42. Howard BE, Lal D. Rhinologic Practice Special Considerations During COVID-19: Visit Planning, Personal Protective Equipment, Testing, and Environmental Controls. Otolaryngol Head Neck Surg. 2020;163: 676–681.

43. Paderno A, Fior M, Berretti G, Schreiber A, Grammatica A, Mattavelli D, et al. SARS-CoV-2 Infection in Health Care Workers: Cross-sectional Analysis of an Otolaryngology Unit. Otolaryngol Head Neck Surg. 2020;163: 671–672.

44. Balasubramanian A, Paleri V, Bennett R, Paleri V. Impact of COVID-19 on the mental health of surgeons and coping strategies. Head Neck. 2020;42: 1638–1644.

45. Lescanne E, van der Mee-Marquet N, Juvanon J-M, Abbas A, Morel N, Klein J-M, et al. Best practice recommendations: ENT consultations during the COVID-19 pandemic. Eur Ann Otorhinolaryngol Head Neck Dis. 2020;137: 303–308.

46. Cho RHW, Yeung ZWC, Ho OYM, Lo JFW, Siu AKY, Kwan WMY, et al. Pearls of experience for safe and efficient hospital practices in otorhinolaryngology—head and neck surgery in Hong Kong during the 2019 novel coronavirus disease (COVID-19) pandemic. Journal of Otolaryngology - Head & Neck Surgery. 2020. doi:10.1186/s40463-020-00427-4

47. Taha MA, Hall CA, Rathbone RF, Corsten LA, Bowie CR, Waguespack PJ, et al. Rhinologic Procedures in the Era of COVID-19: Health-care Provider Protection Protocol. Am J Rhinol Allergy. 2020;34: 451–455.

48. Thamboo A, Lea J, Sommer DD, Sowerby L, Abdalkhani A, Diamond C, et al. Clinical evidence based review and recommendations of aerosol generating medical procedures in otolaryngology - head and neck surgery during the COVID-19 pandemic. J Otolaryngol Head Neck Surg. 2020;49: 28.

49. Lavinsky J, Kosugi EM, Baptistella E, Roithmann R, Dolci E, Ribeiro TK, et al. An update on COVID-19 for the otorhinolaryngologist - a Brazilian Association of Otolaryngology and Cervicofacial Surgery (ABORL-CCF) Position Statement. Braz J Otorhinolaryngol. 2020;86: 273–280.

50. Couloigner V, Schmerber S, Nicollas R, Coste A, Barry B, Makeieff M, et al. COVID-19 and ENT Surgery. Eur Ann Otorhinolaryngol Head Neck Dis. 2020;137: 161–166.

51. Boccalatte LA, Larrañaga JJ, Perez Raffo GM, Teijido CA, García Fornari G, Staneloni MI, et al. Brief guideline for the prevention of COVID-19 infection in head and neck and otolaryngology surgeons. Am J Otolaryngol. 2020;41: 102484.

52. Panuganti BA, Pang J, Califano J, Chan JYK. Procedural precautions and personal protective equipment during head and neck instrumentation in the COVID-19 era. Head Neck. 2020;42: 1645–1651.

53. Park JS, El-Sayed IH, Young VN, Pletcher SD. Development of clinical care guidelines for faculty and residents in the era of COVID-19. Head Neck. 2020;42: 1403–1408.

54. Zhao C, Viana A Jr, Wang Y, Wei H-Q, Yan A-H, Capasso R. Otolaryngology during COVID-19: Preventive care and precautionary measures. Am J Otolaryngol. 2020;41: 102508.

55. Leboulanger N, Sagardoy T, Akkari M, Ayari-Khalfallah S, Celerier C, Fayoux P, et al. COVID-19 and ENT Pediatric otolaryngology during the COVID-19 pandemic. Guidelines of the French Association of Pediatric Otorhinolaryngology (AFOP) and French Society of Otorhinolaryngology (SFORL). Eur Ann Otorhinolaryngol Head Neck Dis. 2020;137: 177–181.

56. Krajewska J, Krajewski W, Zub K, Zatoński T. COVID-19 in otolaryngologist practice: a review of current knowledge. European Archives of Oto-Rhino-Laryngology. 2020. pp. 1885–1897. doi:10.1007/s00405-020-05968-y

57. Cheng X, Liu J, Li N, Nisenbaum E, Sun Q, Chen B, et al. Otolaryngology Providers Must Be Alert for Patients with Mild and Asymptomatic COVID-19. Otolaryngol Head Neck Surg. 2020;162: 809–810.

58. Balakrishnan K, Schechtman S, Hogikyan ND, Teoh AYB, McGrath B, Brenner MJ. COVID-19 Pandemic: What Every Otolaryngologist-Head and Neck Surgeon Needs to Know for Safe Airway Management. Otolaryngol Head Neck Surg. 2020;162: 804–808.

59. Kowalski LP, Sanabria A, Ridge JA, Ng WT, de Bree R, Rinaldo A, et al. COVID-19 pandemic: Effects and evidence-based recommendations for otolaryngology and head and neck surgery practice. Head Neck. 2020;42: 1259–1267.

60. Vukkadala N, Qian ZJ, Holsinger FC, Patel ZM, Rosenthal E. COVID-19 and the Otolaryngologist: Preliminary Evidence-Based Review. Laryngoscope. 2020;130: 2537–2543.

3. SARS-Cov-2 infection in case of pregnancy, breastfeeding, and childbearing potential

61. Rodrigues C, Baía I, Domingues R, Barros H. Pregnancy and Breastfeeding During COVID-19 Pandemic: A Systematic Review of Published Pregnancy Cases. Front Public Health. 2020;8: 558144.

62. Yan H, Ding Y, Guo W. Mental Health of Pregnant and Postpartum Women During the Coronavirus Disease 2019 Pandemic: A Systematic Review and Meta-Analysis. Front Psychol. 2020;11: 617001.

63. Chi J, Gong W, Gao Q. Clinical characteristics and outcomes of pregnant women with COVID-19 and the risk of vertical transmission: a systematic review. Arch Gynecol Obstet. 2020. doi:10.1007/s00404-020-05889-5

64. Amaral WN do, Moraes CL de, Rodrigues APDS, Noll M, Arruda JT, Mendonça CR. Maternal Coronavirus Infections and Neonates Born to Mothers with SARS-CoV-2: A Systematic Review. Healthcare (Basel). 2020;8. doi:10.3390/healthcare8040511

65. Pavlidis P, Eddy K, Phung L, Farrington E, Connolly M, Lopes R, et al. Clinical guidelines for caring for women with COVID-19 during pregnancy, childbirth and the immediate postpartum period. Women Birth. 2020. doi:10.1016/j.wombi.2020.10.015

66. Benski C, Di Filippo D, Taraschi G, Reich MR. Guidelines for Pregnancy Management During the COVID-19 Pandemic: A Public Health Conundrum. Int J Environ Res Public Health. 2020;17. doi:10.3390/ijerph17218277

67. Dimopoulou D, Triantafyllidou P, Daskalaki A, Syridou G, Papaevangelou V. Breastfeeding during the novel coronavirus (COVID-19) pandemic: guidelines and challenges. J Matern Fetal Neonatal Med. 2020; 1–7.

68. Di Toro F, Gjoka M, Di Lorenzo G, De Santo D, De Seta F, Maso G, et al. Impact of COVID-19 on maternal and neonatal outcomes: a systematic review and meta-analysis. Clin Microbiol Infect. 2021;27: 36–46.

69. Hessami K, Romanelli C, Chiurazzi M, Cozzolino M. COVID-19 pandemic and maternal mental health: a systematic review and meta-analysis. J Matern Fetal Neonatal Med. 2020; 1–8.

70. Abou Ghayda R, Li H, Lee KH, Lee HW, Hong SH, Kwak M, et al. COVID-19 and Adverse Pregnancy Outcome: A Systematic Review of 104 Cases. J Clin Med Res. 2020;9. doi:10.3390/jcm9113441

71. Han Y, Ma H, Suo M, Han F, Wang F, Ji J, et al. Clinical manifestation, outcomes in pregnant women with COVID-19 and the possibility of vertical transmission: a systematic review of the current data. J Perinat Med. 2020;48: 912–924.

72. Raschetti R, Vivanti AJ, Vauloup-Fellous C, Loi B, Benachi A, De Luca D. Synthesis and systematic review of reported neonatal SARS-CoV-2 infections. Nat Commun. 2020;11: 5164.

73. Pastick KA, Nicol MR, Smyth E, Zash R, Boulware DR, Rajasingham R, et al. A Systematic Review of Treatment and Outcomes of Pregnant Women With COVID-19-A Call for Clinical Trials. Open Forum Infect Dis. 2020;7: ofaa350.

74. Lubbe W, Botha E, Niela-Vilen H, Reimers P. Breastfeeding during the COVID-19 pandemic - a literature review for clinical practice. Int Breastfeed J. 2020;15: 82.

75. Diriba K, Awulachew E, Getu E. The effect of coronavirus infection (SARS-CoV-2, MERS-CoV, and SARS-CoV) during pregnancy and the possibility of vertical maternal-fetal transmission: a systematic review and meta-analysis. Eur J Med Res. 2020;25: 39.

76. Allotey J, Stallings E, Bonet M, Yap M, Chatterjee S, Kew T, et al. Clinical manifestations, risk factors, and maternal and perinatal outcomes of coronavirus disease 2019 in pregnancy: living systematic review and meta-analysis. BMJ. 2020;370: m3320.

77. Centeno-Tablante E, Medina-Rivera M, Finkelstein JL, Rayco-Solon P, Garcia-Casal MN, Rogers L, et al. Transmission of SARS-CoV-2 through breast milk and breastfeeding: a living systematic review. Ann N Y Acad Sci. 2020. doi:10.1111/nyas.14477

78. Kadir RA, Kobayashi T, Iba T, Erez O, Thachil J, Kazi S, et al. COVID-19 coagulopathy in pregnancy: Critical review, preliminary recommendations, and ISTH registry-Communication from the ISTH SSC for Women's Health. J Thromb Haemost. 2020;18: 3086–3098.

79. Turan O, Hakim A, Dashraath P, Jeslyn WJL, Wright A, Abdul-Kadir R. Clinical characteristics, prognostic factors, and maternal and neonatal outcomes of SARS-CoV-2 infection among hospitalized pregnant women: A systematic review. Int J Gynaecol Obstet. 2020;151: 7–16.

80. Mann A, Dahiya A, Souza LC, Letra A. Considerations for Pregnant Dental and Health Care Workers amid COVID-19. JDR Clin Trans Res. 2020;5: 300–306.

81. Api O, Sen C, Debska M, Saccone G, D'Antonio F, Volpe N, et al. Clinical management of coronavirus disease 2019 (COVID-19) in pregnancy: recommendations of WAPM-World Association of Perinatal Medicine. J Perinat Med. 2020;48: 857–866.

82. Galang RR, Chang K, Strid P, Snead MC, Woodworth KR, House LD, et al. Severe Coronavirus Infections in Pregnancy: A Systematic Review. Obstet Gynecol. 2020;136: 262–272.

83. Walker KF, O'Donoghue K, Grace N, Dorling J, Comeau JL, Li W, et al. Maternal transmission of SARS-COV-2 to the neonate, and possible routes for such transmission: a systematic review and critical analysis. BJOG. 2020;127: 1324–1336.

84. Narang K, Ibirogba E, Elrefaei A, Trad ATA, Theiler R, Picone O, et al. COVID-19 in Pregnancy- A comprehensive summary of current guidelines. Authorea. doi:10.22541/au.158705325.52520124
