# Supplementary material for: Appropriateness for SARS-CoV-2 vaccination for otolaryngologist and head and neck surgeons in case of pregnancy, breastfeeding, or childbearing potential: Yo-IFOS and CEORL-HNS joint clinical consensus statement
Source: Eur Arch Otorhinolaryngol. 2021 Apr 15;278(10):4091–9. doi: 10.1007/s00405-021-06794-6 (PMC8046580; doi:10.1007/s00405-021-06794-6)
Supplement: Supplementary file 5 — Supplementary file5 (PDF 248 KB) [file 405_2021_6794_MOESM5_ESM.pdf]

Appropriateness for SARS-CoV-2 Vaccination for Otolaryngologist and Head and Neck Surgeons in case of Pregnancy, Breastfeeding or Childbearing potential: Yo-IFOS and CEORL-HNS joint clinical consensus statement

Journal: European Archives of Oto-Rhino-Laryngology

Authors: Saibene Alberto Maria, et al.

Correspondence to: Alberto Maria Saibene, Otolaryngology Unit - ASST Santi Paolo e Carlo. Via Antonio di Rudinì, 8 - 20142 - Milan, Italy. Phone: +39 02 8184 4249. Fax: +39 02 5032 3166. Mail: alberto.saibene@gmail.com

Online resource 5: Statements and their evolution through the Delphi process

Item No.	I version	First Delphi Results	Decision	II version	Second Delphi Results	Final Decision
1	Otolaryngology and head and neck surgery represent specialties at high risk of SARS-CoV-2 infection	consensus	accepted in CCS	N/A	N/A	included in CCS in its original version
2	The prevention measures and the correct use of personal protective equipment can mitigate but not completely remove the specialty-related SARS-CoV-2 infection risk	no consensus (mean 7.95)	revise	Although preventive measures and use of full personal protective equipment has been demonstrated to prevent SARS-CoV-2 infection, due to environmental, behavioral, and practical contingencies, the specialty-related risk of infection can be minimized but not completely removed	consensus	included in CCS
3	COVID-19 infection during pregnancy may be associated with an increased risk of preterm delivery and fetal growth restriction and more severe forms of COVID-19 illness for the mother	no consensus (mean 7.34)	revise	Pregnant people with COVID-19 might be at increased risk of adverse pregnancy outcomes compared with pregnant women without COVID-19 and, although chances for severe health effects are low, pregnant people with COVID-19 have an increased risk of severe illness compared with non-pregnant women of reproductive age	no consensus (mean 7.33)	excluded from CCS
4	Albeit in absence of specific trials and evidence,	no	revise	Though the recently developed SARS-CoV-2	consensus	included in

	the recently developed SARS-CoV-2 mRNA vaccines do not seem to show a risk profile for complication for the mother-baby dyad during pregnancy and breastfeeding	consensus (mean 7)		mRNA vaccines do not seem to show a risk profile for complication for the mother-baby dyad during pregnancy and breastfeeding, we have no experimental data in this population on which no trial has been conducted and no long-term evaluation is available		CCS
5	All pregnant, breastfeeding or fertile female otolaryngologists and head and neck surgeons considering a COVID-19 vaccine should have access to up-to-date information about the safety and efficacy of the vaccine for the mother-baby dyad, including clear information about data and evidence that are not available yet for this specific population	consensus	accepted in CCS	N/A	N/A	included in CCS in its original version
6	All pregnant otolaryngologists and head and neck surgeons who are active in clinical practice should be given the opportunity to receive rapidly the SARS-CoV-2 vaccine	no consensus (mean 7.68)	revise	All pregnant otolaryngologists and head and neck surgeons who are active in clinical practice should be given the opportunity to receive the SARS-CoV-2 vaccine rapidly, provided the choice is free, individual, and informed and assisted by a health professional to individually assess the benefits and risks according to each case	strong consensus	included in CCS
7	All pregnant otolaryngologists and head and neck surgeons who are active in clinical practice are encouraged to receive the SARS-CoV-2 vaccine	no consensus (mean 6.56)	revise	All pregnant otolaryngologists and head and neck surgeons who are active in clinical practice may be encouraged to receive the SARS-CoV-2 vaccine rapidly, provided the choice is free, individual, and informed and assisted by a health professional to individually assess the benefits and risks according to each case	no consensus (mean 7.5)	excluded from CCS
8	All breastfeeding otolaryngologists and head and neck surgeons who are active in clinical practice or expect to resume clinical practice before stopping breastfeeding should be given the opportunity to receive the SARS-CoV-2 vaccine	no consensus (mean 7.81)	revise	All breastfeeding otolaryngologists and head and neck surgeons should be given the opportunity to receive the SARS-CoV-2 vaccine, provided the choice is free, individual, and informed and assisted by a health professional to individually assess the benefits and risks according to each case	near consensus (mean 8.17)	excluded from CCS
9	All breastfeeding otolaryngologists and head and neck surgeons who are not active in clinical practice and don’t expect to resume clinical practice before stopping breastfeeding, should	no consensus (mean 6.4)	removed from Delphi process	N/A	N/A	excluded from CCS in its original

	wait for the end of breastfeeding before receiving the SARS-CoV-2 vaccine and use appropriate contraception prior to vaccination and up to 2 months after receiving the second vaccine dose					version
10	All non-pregnant and non-breastfeeding otolaryngologists and head and neck surgeons of childbearing potential who opt for receiving the SARS-CoV-2 vaccine should use appropriate contraception prior to vaccination and up to 2 months after receiving the second vaccine dose.	no consensus (mean 6.2)	removed from clinical consensus statement	N/A	N/A	excluded from CCS in its original version
11	All pregnant and breastfeeding otolaryngologists and head and neck surgeons who decline vaccination should be strongly stimulated to keep in mind prevention measures such as hand washing, physical distancing, wearing a mask, and using proper personal protection devices	consensus	accepted in CCS	N/A	N/A	included in CCS in its original version
12	The use of adequate personal protective equipment against SARS-CoV-2 remains strongly recommended for otolaryngologist and head and neck surgeons who received the SARS-CoV-2 vaccine	strong consensus	accepted in CCS	N/A	N/A	included in CCS in its original version
13	Since prenatal maternal stress is also associated with neurodevelopmental disorders among exposed offspring, all pregnant otolaryngologists and head and neck surgeons should assess and balance the psychological burden imposed by the risk of SARS-CoV-2 infection to that of receiving the SARS-CoV-2 vaccine.	no consensus (mean 7.59)	revise	Since prenatal maternal stress is also associated with neurodevelopmental disorders among exposed offspring, all pregnant otolaryngologists and head and neck surgeons should take into account in the informed and assisted decision to take the SARS-Cov-2 vaccine not only the infection risk but also the psychological burden imposed by the risk of SARS-CoV-2 infection, adequately balanced to that of receiving the SARS-CoV-2 vaccine	no consensus (mean 7.67)	excluded from CCS
